# Minimally overlapping words for sequence similarity search

**DOI:** 10.1093/bioinformatics/btaa1054

**Published:** 2020-12-21

**Authors:** Martin C Frith, Laurent Noé, Gregory Kucherov

**Affiliations:** btaa1054-aff1 Artificial Intelligence Research Center, AIST, Tokyo, Japan; btaa1054-aff2 Graduate School of Frontier Sciences, University of Tokyo, Chiba, Japan; btaa1054-aff3 AIST-Waseda University CBBD-OIL, AIST, Tokyo, Japan; btaa1054-aff4 CRIStAL UMR9189, Université de Lille, Villeneuve d’Ascq, France; btaa1054-aff5 LIGM, CNRS, Université Gustave Eiffel, Marne-la-Valleé, France; btaa1054-aff6 Skolkovo Institute of Science and Technology, Moscow, Russia

## Abstract

**Motivation:**

Analysis of genetic sequences is usually based on finding similar parts of sequences, e.g. DNA reads and/or genomes. For big data, this is typically done via ‘seeds’: simple similarities (e.g. exact matches) that can be found quickly. For huge data, sparse seeding is useful, where we only consider seeds at a subset of positions in a sequence.

**Results:**

Here, we study a simple sparse-seeding method: using seeds at positions of certain ‘words’ (e.g. ac, at, gc or gt). Sensitivity is maximized by using words with minimal overlaps. That is because, in a random sequence, minimally overlapping words are anti-clumped. We provide evidence that this is often superior to acclaimed ‘minimizer’ sparse-seeding methods. Our approach can be unified with design of inexact (spaced and subset) seeds, further boosting sensitivity. Thus, we present a promising approach to sequence similarity search, with open questions on how to optimize it.

**Availability and implementation:**

Software to design and test minimally overlapping words is freely available at https://gitlab.com/mcfrith/noverlap.

**Supplementary information:**

Supplementary data are available at *Bioinformatics* online.

## 1 Introduction

### 1.1 Backgound and aim

Sequence similarity search remains fundamental in bioinformatics. It has a basic tradeoff between sensitivity and computational cost (time and memory use). We present here an approach that advances the Pareto frontier in the low-cost, low-sensitivity region: in other words, we show how to achieve very low cost with not-so-low sensitivity. This is useful for: huge sequence data where minimizing computational cost is essential; moderately large data requiring fast analysis, e.g. in clinical applications; and interactive-speed analysis of moderate-size data. This is timely because large datasets are becoming more ubiquitous, e.g. whole-genome sequencing, genomes or transcriptomes from thousands of single cells, or deep sequencing of DNA from an environment, such as seawater. While many methods are optimized for human genomes (3 Gb), some important genomes are larger, e.g. wheat (17 Gb) and oat (12 Gb). We do not describe an implementation, but rather a new theoretical approach that could be used in various sequence search tools: we have implemented it in LAST (http://last.cbrc.jp/). In order to explain our approach, we first review alignment seeds and sparse seeding.

### 1.2 Alignment seeds and sparse seeding

Finding similar sequences, in large data, is typically done via ‘seeds’: simple similarities that can be found quickly. The simplest type of seed is exact matches of a given length, e.g. 10 letters for DNA. The seed length affects the sensitivity and run time: shorter seeds are more sensitive, but find more hits that must then be checked. By lengthening the seeds, we can arbitrarily reduce the run time of the downstream steps, but not the time and memory usage for finding the seeds.

An alternative way to reduce time and/or memory use is sparse seeding. The simplest way is to only use seeds starting at every *n*th position in one of the two sequences being compared (if we only use seeds at every *n*th position in *both* sequences, the sensitivity will be poor. For example, we cannot find identical segments starting at coordinate *x* in the first sequence and *x *+* *1 in the second sequence). Sparse seeding reduces sensitivity, but we could then increase the sensitivity by shortening the seeds. This raises the prospect of reducing run time and/or memory use without loss of sensitivity.

An intriguing idea is to achieve sparsity by selecting seeds starting at positions of certain words. For example, if we only use seeds starting with ‘a’ (Paul Horton, personal communication), we achieve 4-fold sparsity in both sequences without huge loss of sensitivity. We can imagine more complex variants, e.g. use seeds starting with any of these words: ac, at, gc, gt. One version of this is seeding at (arbitrary) words that hash to 0 mod n, for some hash function and some positive integer *n* (Manber, 1994; Schleimer *et al.*, 2003).

### 1.3 Summary of our contribution

We improve word-based seeding, by showing that some word sets are better than others: so we can benefit from using *designed* rather than *arbitrary* word sets. Specifically, it is better to use words with minimal overlap (e.g. ac, at, gc, gt *cannot* overlap). The reason, briefly, is that in a random sequence, minimally overlapping words occur with more uniform spacing, i.e. they are anti-clumped or under-dispersed; equivalently, their number of occurrences has lower variance. We show evidence that this sparse-seeding approach is superior to a currently popular alternative: minimizers. Finally, we show that word-based seeding can be naturally unified with *inexact* seeds (spaced and subset seeds), further boosting sensitivity. The remainder of this introduction reviews further background important for this study.

### 1.4 Minimizers

The minimizer method is a bit more complex (Roberts *et al.*, 2004; Schleimer *et al.*, 2003). First, we must define an ordering of the positions in a sequence, e.g. by alphabetic order of the suffix starting at each position. Then, we identify all positions that are the minimum in *any* window of *w* consecutive positions (e.g. *w* = 7). Only seeds starting at these positions are used.

Various orderings can be used, e.g. compare two suffixes using order c <a <t <g at odd-numbered bases and g <t <a <c at even-numbered bases, so that cgcg… is the minimum possible suffix (Roberts *et al.*, 2004). The resulting degree of sparsity is not obvious, and it depends on the ordering (Marçais *et al.*, 2017). Typically, a fraction 2/(w+1) of positions is selected (Schleimer *et al.*, 2003).

Another related idea is universal *k*-mer hitting sets (Orenstein *et al.*, 2017). This means a set of length-*k* words, such that every possible length-*L* sequence contains at least one of the words. Recent studies have defined minimizer orderings based on universal *k*-mer hitting sets, resulting in high sparsity for a given *w* (Marçais *et al.*, 2017, 2018; Orenstein *et al.*, 2017).

Minimizers have been described as ‘a central recent paradigm’ (Orenstein *et al.*, 2017): they have been widely used for sparse seeding (e.g. Jain *et al.*, 2018; Li, 2018) and other applications (e.g. Deorowicz *et al.*, 2015; Li *et al.*, 2013; Wood and Salzberg, 2014).

### 1.5 Spaced and subset seeds

So far, we have considered exact-match seeds, but inexact seeds are also used. One variant is spaced seeds, which allow mismatches at some fixed positions in the seed (e.g. positions 3 and 5 out of 9). Spaced seeds are often superior to exact seeds (Ma *et al.*, 2002), because their hits are less concentrated in overlapping clumps. Thus, spaced seeds have been designed by minimizing their ‘overlap complexity’ (Ilie and Ilie, 2007), which is similar to minimizing the variance in number of hits (Hahn *et al.*, 2016).

Subset seeds are a further generalization: they allow *some* mismatches (e.g. a ↔g and c ↔t) at fixed positions (Noé and Kucherov, 2004). This is useful for DNA, because a ↔g and c ↔t substitutions (termed ‘transitions’) are often more frequent than the other types of substitution (‘transversions’). Transition seeds have also been designed for use with every-*n*th sparsity (Frith and Noé, 2014).

### 1.6 Repeats

Natural DNA has many repeats, which are the main difficulty for similarity search. For example, a primate genome may have a million Alu elements, so naive comparison of such genomes yields an unmanageable 10^12^ significant similarities. Our practical aim cannot be to find all significant similarities, but rather orthologs and/or strongest similarities. In any case, a seeding method must avoid getting too many repetitive seeds. One solution is to omit high-frequency seeds, another is to use variable-length seeds that are made longer until they are sufficiently rare (Csűrös, 2004; Kielbasa *et al.*, 2011).

### 1.7 Non-overlapping words

Since we are interested in minimally overlapping words, let us consider non-overlapping words. A basic question is: what is the maximum possible number of non-overlapping words of some length *k*? That is, given an alphabet of size *a* (so there are *a^k^* possible words), what is the maximum possible number of words where no proper prefix of any word equals a proper suffix of any word? This seems hard to answer in general (Blackburn, 2015).

The following construction has been suggested for getting a large number of non-overlapping words (Blackburn, 2015). Divide the alphabet into two subsets, e.g. {*a*} and {*c*, *g*, *t*}, and choose a prefix length *j* (0<j<k). These words have no overlaps: words whose first *j* letters are from the first subset, whose (j+1)^th^ and *k*th letters are from the second subset, and whose letters between *j *+* *1 and *k* have no run of ≥j letters from the first subset.

## 2 Materials and methods

### 2.1 Mean and variance

Given a set of length-*k* words, let us consider their occurrence in a random i.i.d. length-*s* sequence. Define *I_j_* to be 1 if any of the words occurs starting at position *j*, else 0. The number of occurrences is $X=I_{1}+I_{2}+\cdots +I_{s-k+1}$, and the expected number is:

(1) E[X]=(s−k+1)p,

where *p* is the total probability of any of the words occurring at a given position. The variance in occurrence number is:

(2) $$\text{Var}[X]=E[X^{2}]-E{\left[X\right]}^{2},$$

where

(3) $$E[X^{2}]=E[{\left(I_{1}+I_{2}+\cdots +I_{s-k+1}\right)}^{2}]$$

(4) $$=\sum_{i,j}E[I_{i}I_{j}].$$

If we define l=|i−j|, then

(5) $$E[I_{i}I_{j}]=\{\begin{array}{cc} p & \text{if}\;l=0\\ \sum_{V,W}B_{VW}^{k-l}P_{V}^{l}P_{W}^{k} & \text{if}\;0<l<k\\ p^{2} & \text{if}\;l\geq k, \end{array}$$

where $B_{VW}^{m}$ is defined to be 1 if the length-*m* suffix of word *V* equals the length-*m* prefix of word *W*, else 0. Also, $P_{W}^{n}$ is the product of probabilities of the first *n* letters in word *W*. Thus, assuming that s≥2k−2 (see the Supplementary Material):

(6) $$\begin{array}{c} \text{Var}[X]=(s-k+1)p\\ +2\sum_{V,W}\sum_{l=1}^{k-1}\;[(s-k+1-l)B_{VW}^{k-l}P_{V}^{l}P_{W}^{k}]\\ -[(2k-1)s-(3k-1)(k-1)]p^{2}. \end{array}$$

For circular sequences, the formulas are simpler (assuming s≥2k−1):

(7) E[X]=sp

(8) $$\text{Var}[X]=s[p-(2k-1)p^{2}+2\sum_{V,W}\sum_{l=1}^{k-1}\left(B_{VW}^{k-l}P_{V}^{l}P_{W}^{k}\right)].$$

These formulas also apply to linear sequences when s≫k. With these formulas, the variance-to-mean ratio (VMR), also called index of dispersion, is independent of the sequence length. The formulas also simplify for linear sequences with s=2k−1 (the smallest *s* where all kinds of pairwise overlap contribute):

(9) E[X]=kp

(10) $$\text{Var[X] = }k_{p}\;-\;k^{2}p^{2}+2\sum_{V,W}\sum_{l=1}^{k-1}\;[(k-l)B_{VW}^{k-l}P_{V}^{l}P_{W}^{k}].$$

### 2.2 Simulated sequences

To test homology detection, DNA sequences were simulated with the T92 model of evolution (Tamura, 1992). This model has three input parameters: gc-content, transition/transversion rate ratio *κ* and PAM substitution distance (Table 1).

**Table 1.** Parameters of the T92 DNA model

PAM	*κ*	%g + c	%identity	Transitions per transversion
20	1	50	82	0.5
50	1	50	64	0.5
20	3	50	83	1.4

For each test, 100 000 pairs of DNA sequences were simulated. The default parameters, unless specified otherwise, are: %g + c=50, *κ*=1 (unbiased), PAM=20, sequence length = 100. A seeding method was deemed to find a pair of sequences if it found at least one match at identical coordinates of the pair.

To test specificity, two unrelated length-10^6^ sequences were generated, and the number of seed pair matches counted. This is a proxy for the computational cost of checking all the seed hits.

## 3 Results

### 3.1 Non-overlapping DNA words

The maximum possible number of non-overlapping DNA words, for word length *k* = 2 to 6 (Table 2), was found by brute-force clique search (Konc and Janežič, 2007). For *k* < 6, Blackburn’s construction (Blackburn, 2015) achieves this maximum. For *k* = 2, a maximum set is ry (r = a or g, y = c or t). In general, abb… (b = any base except a) is a good way to get non-overlapping words, and a nice generalization of Horton’s idea.

**Table 2.** Non-overlapping DNA words

Word	Constructed		Maximum
length	Words	Number	number
2	ry	4	4
3	abb	9	9
4	abbb	27	27
5	abbbb	81	81
6	abbbbb	243	251

*Note*: r={a,g}; y={c,t}; b={c,g,t}.

### 3.2 Every-*n*th sparsity

**Fig. 1.**
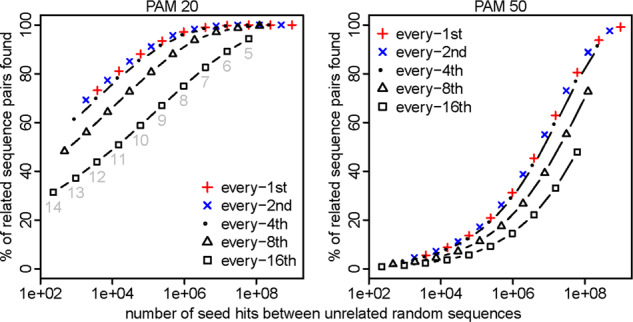
Sensitivity (*y-*axis) and spurious hit count (*x*-axis) for exact-match seeds with every-*n*th sparsity. Sensitivity was measured on sequence pairs with PAM distance 20 (left panel) or 50 (right panel). Seed lengths 5–14 were tested, shown in gray in the left panel

We first tested every-*n*th sparsity (only using seeds starting at every *n*th position in one of the two sequences being compared), with exact-match seeds. We defined ‘sensitivity’ as % of sequence pairs with ≥1 seed match at homologous positions. As expected, if we increase sparsity without changing the seed length, both sensitivity and random hit count decrease (Fig. 1). If we then shorten the seeds, the sensitivity and random hit count increase. The important result is that higher sparsity has lower sensitivity *for a given random hit count*. The exception is *n* = 2, which is no worse than *n* = 1, indeed giving us something for nothing: sparsity at no cost.

A plausible explanation for why *n* = 1 is not better than *n* = 2 is that highly overlapping seeds provide little independent information. This is also why spaced seeds are better than exact-match seeds. Thus, it would be interesting to compare *n* = 1 to *n* = 2 using optimized spaced/subset-seed patterns: this was done previously, and *n* = 2 was worse (Frith and Noé, 2014).

### 3.3 Sparsity via words

**Fig. 2.**
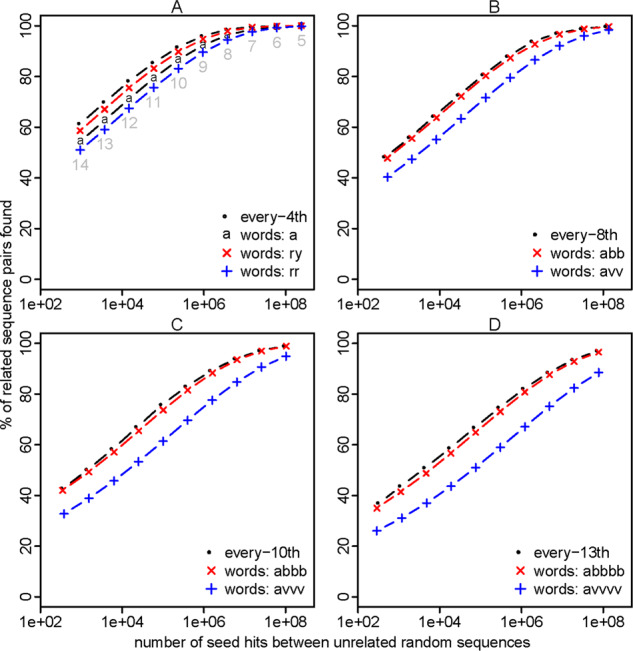
Sensitivity (y-axis) and spurious hit count (x-axis) for exact-match seeds with every nth or word-based sparsity (A, B, C, D). Sensitivity was measured on sequence pairs with PAM distance 20. Seed lengths 5–14 were tested, as shown in (**A**)

Let us now see how seeds starting with ‘a’ compare to every-4th seeding. For a given seed length, the random hit counts are the same (as expected), but seeds starting with ‘a’ have lower sensitivity (Fig. 2A). This is not too surprising, because every-4th seeding is sparse in just one sequence, but seeds starting with ‘a’ are sparse in both sequences. Seeds starting with ry also have the same random hit counts, and their sensitivity is closer to (but still less than) that of every-4th seeds. On the other hand, seeds starting with rr have worse sensitivity. This supports the idea that non-overlapping words are good and highly overlapping words are bad.

Seeds starting with abb have a sparsity factor of $4^{3}/3^{2}\approx 7.1$, and they perform slightly worse than every-8th seeds (Fig. 2B). On the other hand, they perform better than seeds starting with avv (v = any base except t). Seeds starting with abbb (sparsity 9.5) or abbbb (sparsity 12.6) show similar results (Fig. 2C and D), confirming the advantage of non-overlapping words.

### 3.4 Minimal-variance words

We can perhaps do better by using longer words with some overlap. Seeds starting with ry are the same as seeds starting with ryn (where n is any base), so it may be better to replace ryn with a less-overlapping set of length-3 words.

It is not obvious how best to quantify ‘amount of overlap’, but one idea is to use variance of occurrence number in random sequences. Let us try these two measures of overlap: VMR1 [from Equations (7) and (8)] and VMR2 [from Equations (9) and (10)].

It is also unclear how to find a set of words that minimizes VMR1 or VMR2, because the number of possible sets is enormous. Brute-force search is feasible if we restrict ourselves to a 2-letter ry alphabet.

**Fig. 3.**
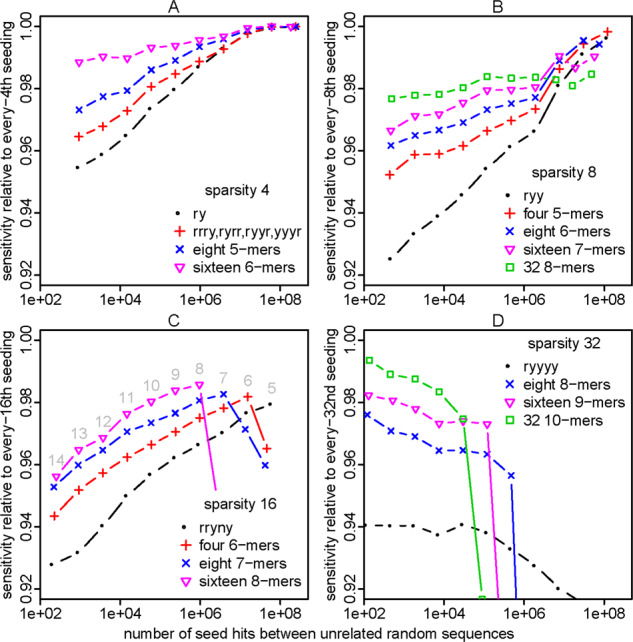
Sensitivity (y-axis) and spurious hit count (x-axis) for exact-match seeds with word-based sparsity (A, B, C, D). Sensitivity was measured on sequence pairs with PAM distance 20. Seed lengths 5–14 were tested, as shown in (**C**). In this figure, the sensitivity is shown relative to every-*n*th sparsity: (% of related sequence pairs found by word-restricted seeds)/(% of related sequence pairs found by every-*n*th seeds)

Such words can indeed boost sensitivity. For example, the words rrry, ryrr, ryyr, yyyr have lower VMR2 than rynn (Table 3), and seeds starting at these words have better sensitivity (Fig. 3A). We can do even better with 8 length-5 words, and better still with 16 length-6 words (Table 3 and Fig. 3A).

**Table 3.** Variance-to-mean ratios

Words	VMR1	VMR2
*sparsity 4*		
ry	**0.25**	**0.5**
ryn	**0.25**	**0.417**
rynn	**0.25**	0.375
rrry, ryrr, ryyr, yyyr	**0.25**	**0.344**
rrrry, rryrr, ryryr, ryyrr,		
ryyry, ryyyy, yyyrr, yyyry	**0.125**	**0.25**
rrrrry, rryrry, rryryy, ryrrrr,		
ryrrry, ryryry, ryyrrr, ryyrry,		
ryyryr, ryyryy, ryyyry, ryyyyy,		
yryrry, yyyrrr, yyyrry, yyyyry	**0.0938**	**0.188**
*sparsity 8*		
ryy	**0.375**	**0.625**
rrrry, yrrry, yrryy, yryyy	**0.25**	**0.45**
rrrrry, yrrrry, yrrryr, yrrryy,		
yrryry, yrryyy, yyryry, yyryyy	**0.188**	**0.372**
rrryrrr, rrryryr, rryrryr, rryyrrr,		
rryyrry, rryyryr, rryyyrr, rryyyyr,		
ryryyrr, ryyyryr, ryyyyyr, ryyyyyy,		
yryyryr, yryyyrr, yryyyyr, yyryyrr	**0.176**	**0.329**
rrrrrrry, rryrrryy, ryrrrryr, ryrrrryy,		
ryrrryry, yrrrrrry, yrrrrryr, yrrrrryy,		
yrrryrry, yrryrryr, yrryrryy, yryrrryy,		
yryrryry, yryrryyr, yryrryyy, yryryryy,		
yyrrrryr, yyrrrryy, yyrrryry, yyrrryyr,		
yyrrryyy, yyrryryr, yyrryryy, yyrryyry,		
yyrryyyr, yyrryyyy, yyryryyr, yyryryyy,		
yyryyryy, yyyryyyr, yyyryyyy, yyyyyyyr	0.151	0.281

*Note*: Bold values are known to be the minimum possible, for that sparsity and word length.

We attempted to find best-possible sets of minimal-variance words, for a useful range of sparsities (Table 3 and Supplementary Table S1). For more than ∼16 words, our brute-force search was too slow, so we switched to a heuristic search method (simulated annealing) that does not guarantee to find the minimum possible VMR. On one hand, we successfully obtained high-sensitivity word sets (Fig. 3); on the other hand, we found that decreasing VMR does not always increase sensitivity (Supplementary Fig. S2). Thus, a better criterion for choosing a set of words is still to be designed. Another limitation is that longer words perform badly when they exceed the seed length (e.g. right-hand part of Fig. 3C). In short, we provide useful word sets for sparse seeding, but there is scope for further understanding and improvement.

### 3.5 Minimizers

We next tested minimizers, with three orderings:

- Alphabetic order.
- cg-order, where cgcg… is the minimum sequence. This is representative of methods that have been used in practice (Marçais *et al.*, 2017).
- abb-order. This is a novel ordering, inspired by non-overlapping abb… words. It compares two suffixes using order a <c <g <t at the first position and t=g=c <a at all subsequent positions.

**Fig. 4.**
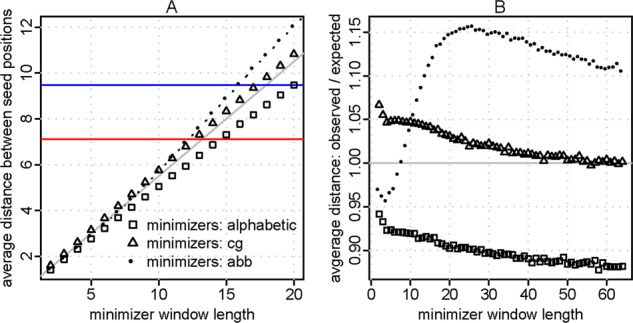
Sparsity of minimizers, with three orderings. Red line: sparsity of abb words. Blue line: sparsity of abbb words. The diagonal gray line in (**A**), and the horizontal gray line in (**B**), show the expected minimizer sparsity (w+1)/2

Let us first see the sparsity (average distance between seed start coordinates) of these orderings. Alphabetic minimizers have the lowest sparsity (highest density) for a given window length *w*, and cg minimizers have higher sparsity (Fig. 4), as reported previously (Marçais *et al.*, 2017). Interestingly, abb minimizers have even higher sparsity for *w* > 10.

**Fig. 5.**
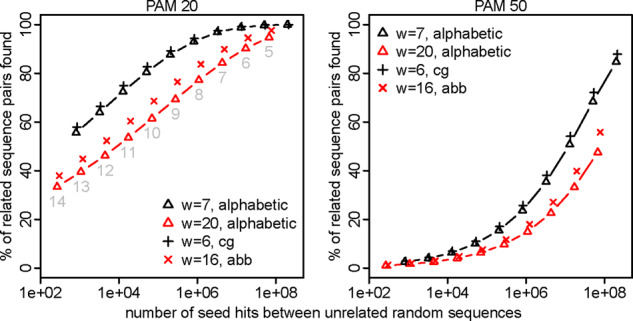
Sensitivity (*y*-axis) and spurious hit count (*x*-axis) for exact-match seeds at minimizer positions. ‘w’ means window length. Seed lengths 5–14 were tested, shown in gray in the left panel

Now let us see the sensitivity of these minimizers. Taking alphabetic minimizers as an example, if we increase the window size *w* without changing the seed length, the sensitivity and random hit rate both decrease (Fig. 5), as expected. If we then shorten the seeds, the sensitivity and random hit rate increase. Overall, higher *w* results in lower sensitivity for a given random hit count.

To fairly compare the three kinds of minimizer, we should compare them using *different* window sizes that achieve the *same* sparsity. Based on Figure 4A, alphabetic minimizers with *w* = 7 are comparable to cg minimizers with *w* = 6, and alphabetic minimizers with *w* = 20 are comparable to abb minimizers with *w* = 16. Comparing them thus, cg and abb minimizers are better than alphabetic minimizers (Fig. 5). This supports the idea that higher sparsity for a given *w* improves homology search, which does not seem to have been clearly shown before.

### 3.6 Minimizers versus words

**Fig. 6.**
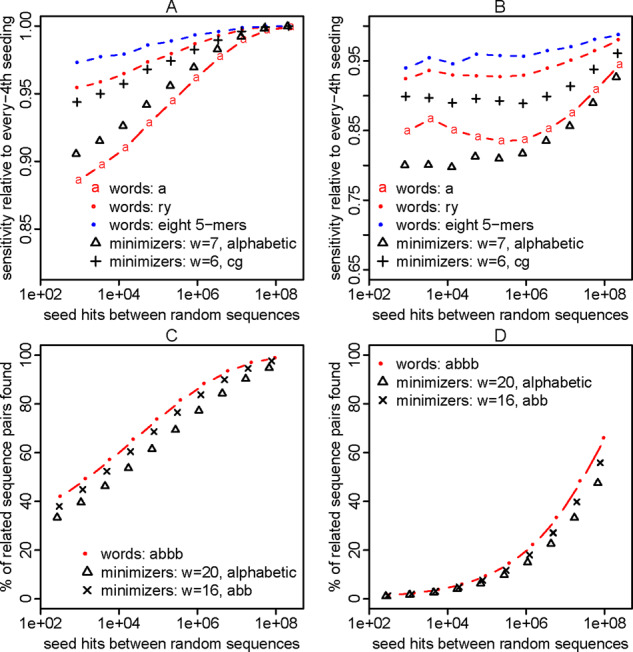
Sensitivity (*y*-axis) and spurious hit count (*x*-axis) for exact-match seeds at either word positions or minimizer positions. Seed lengths 5–14 were tested. Sensitivity was measured on sequence pairs with PAM distance 20 (**A**, **C**) or 50 (**B**, **D**)

To fairly compare minimizers with words, we should use minimizer window sizes that produce the same sparsity as the words. Figure 6A and B compares words to minimizers with slightly lower sparsity (higher density), giving an unfair advantage to the minimizers. Seeds starting with ‘a’ perform worse than alphabetic minimizers for PAM distance 20 (Fig. 6A), but better for PAM distance 50 (Fig. 6B). On the other hand, seeds starting at non-overlapping (ry) or minimum-variance words perform better than alphabetic or cg minimizers, at both PAM distances.

Figure 6C and D compares words to minimizers with about the same sparsity. Seeds at positions of abbb perform slightly better than alphabetic or abb minimizers. In more detail: for a given seed length, the minimizers have worse sensitivity but slightly better specificity.

Next, we compared word-based seeding to the minimizer scheme of the minimap2 software (Li, 2018). This scheme uses exact-match seeds of length 15, with minimizer window *w* = 10, and an ordering from a particular hash function applied to each 15-mer. The expected density is 2/(w+1)=0.1818, but we empirically found a slightly higher density, 0.185–0.188, in both random and real sequences. We compared this to 12 length-6 ry words (density $12/2^{6}=0.1875$) that minimize VMR2: rrrrry, rryrrr, rryrry, rryyrr, rryyry, ryryrr, ryyyrr, ryyyry, ryyyyr, yryyrr, yryyry and yyyyyr.

**Fig. 7.**
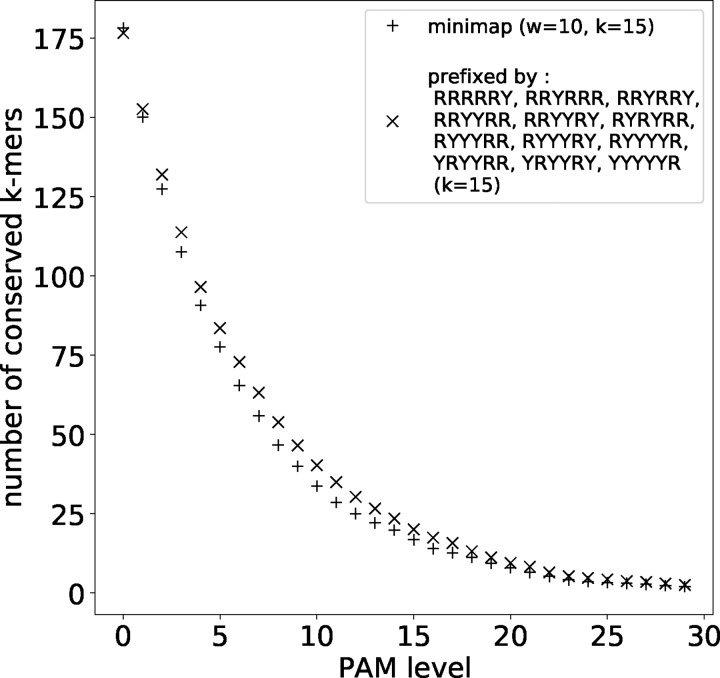
Sensitivity (*y*-axis) at different evolutionary distances (*x*-axis), for minimap seeds and word-based seeds. Here, ‘sensitivity’ is the average number of conserved seeds over 1000 pairs of length-1000 sequences from human chromosome 22

For this test, random fragments of size 1000 were drawn from human (GRCh38) chromosome 22, then mutated by the PAM process, and the number of conserved 15-mer seeds was counted. At PAM distance 0, minimap has more seeds, i.e. higher density (Fig. 7). Nevertheless, at PAM distance ≥1, minimap has fewer conserved seeds.

### 3.7 Unification with subset seeds

A straightforward generalization of subset seeds incorporates word-restricted seeding. Recall that subset seeds allow some mismatches (e.g. a ↔g and c ↔t) at fixed positions. More precisely, each position in the seed specifies an equivalence relation on the letters of the sequence alphabet, e.g. {{a,g},{c,t}} (Roytberg *et al.*, 2009). Our generalization is: each position specifies an equivalence relation on *some* letters of the sequence alphabet.

Such a seed can be described by a *pattern*, such as: ANNRYrn@y. This specifies seeds of length 9. Positions with A (in this case, the first position) allow a: a matches only. Positions with *N* allow any match. Positions with *n* allow any match or mismatch. Positions with *R* allow purine matches only: a: a or g: g. Positions with *r* allow purine matches or mismatches: a: a, g: g, a: g, g: a. Positions with *Y* or *y* likewise allow pyrimidines (c and t). Finally, positions with @ allow any match or transition.

The *sparsity* and *weight* of a seed pattern refer to hit probabilities in random i.i.d. sequences. Consider a seed pattern *U* with length *s*, and two random sequences, *A* and *B*, each of length *s*. Define $p(A\overset{U}{∼}B)$ as the probability that *A* and *B* match according to *U*. Sparsity means rarity of compatible positions in one random sequence, and can be defined as: $1/p(A\overset{U}{∼}A)$. Weight indicates unlikelihood of a chance match to a compatible position: $\text{log}\;p(A\overset{U}{∼}B|A\overset{U}{∼}A)/\;\text{log}\;p(A_{1}=B_{1})$. The denominator is just a scale factor, to make the weight of an exact-match seed equal its length, as is traditional.

The hard problem is to design good seed patterns for finding sequences related by a given PAM distance, transition/transversion bias, etc. Fortunately, the seed design software Iedera already allowed this kind of generalized subset seed (Kucherov *et al.*, 2006). Here, we used it to design seeds for PAM =20 and *κ* =3. To constrain the search space in this preliminary study, we only considered patterns based on ry, i.e. having one *R* or *r* and one *Y* or y, and likewise patterns based on ryy. The only other pattern symbols allowed were *N*, @, and up to 5 *n*s. Up to 10 transition-tolerant positions other than *n* were allowed. The resulting patterns are in Table 4. Many other patterns are equally good; we broke ties by preferring ones that start with RY or RYY.

**Table 4.** Seed patterns designed by Iedera for PAM 20, *κ* =3, alignment length 64

Weight	Pattern
*ry-based seeds: sparsity 4*	
5	RYNN@@
6	RY@@@@NN
7	RYN@@@@NN
8	RYN@@@@NNN
9	RYN@@@@@@NNN
10	RYN@@@nnNN@@@NN
11	RYN@@@nnNN@@@NNN
12	RYN@@@@NNnn@@@@NNN
13	RYN@@@@NN@nn@@@NNNN
14	RYN@@@@NN@nn@@@@NNNN@
*ryy-based seeds: sparsity 8*	
5	RYY@@@@
6	RYYN@@@@
7	RYYN@@@@@@
8	RYYNN@@@@@@
9	RYY@@@@@@@@NN
10	RYY@@@@@@@@NNN
11	RYYN@@@@@@@@NNN
12	RYYN@@@@@@@@@@NNN
13	RYYN@@@@@@@@@@NNNN
14	RYYN@@@@@@@@@@NNNNN

One notable result is that *n* positions are useful in the ry-based seeds, but not the ryy-based seeds. This is presumably because *n* positions make overlapping seeds more independent, but sparser seeds have fewer overlaps.

**Fig. 8.**
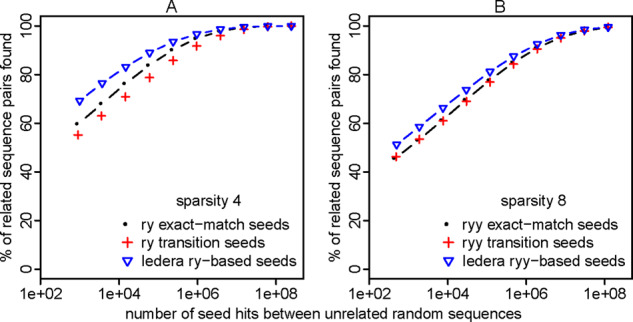
Sensitivity (*y*-axis) and random hit count (*x*-axis) of seeding methods, for sequences with transition/transversion bias (*κ* =3) and PAM distance 20. Seed weights 5–14 were tested. ‘Transition seeds’ allow transition substitutions at all positions

As expected, these seed patterns are good for finding sequences that are related by PAM = 20 and *κ* =3 (Fig. 8).

## 4 Discussion

### 4.1 When to use sparsity

The aim of seeding methods is to maximize sensitivity while minimizing computational cost (time and memory). Computational cost has two parts: the cost of finding seed matches (*c*_1_) and the cost of processing them (*c*_2_). Sparsity need not reduce sensitivity, if the seeds are shortened, but it usually increases random seed hits (i.e. *c*_2_) for a given sensitivity (Fig. 1). A notable exception is exact-match seeds and every-*n*th sparsity with small *n* (e.g. *n* = 2), which does not increase random hits for a given sensitivity (Fig. 1). Typically, however, sparsity is beneficial only when long (or rare) seeds do not sufficiently reduce the computational cost.

### 4.2 When to use every-*n*th sparsity

Every-*n*th sparsity has better sensitivity per random hits (*c*_2_) than either minimizers or word-restricted seeds, see also Almutairy and Torng (2018). So it should be preferred unless its *c*_1_ is significantly worse. It achieves sparsity in just one of the two sequence datasets being compared, which is appropriate for comparing a huge dataset to a moderate-size dataset, e.g. many DNA reads to a moderate-size genome. It *might* be appropriate for comparing DNA reads to a human genome.

Sparsity in both datasets, with minimizers or word-restricted seeds, is appropriate for ‘huge-versus-huge’ comparisons. A typical example is aligning DNA reads to each other in order to assemble them, which was a major motivation for minimizers (Roberts *et al.*, 2004). Other examples are searching DNA sequences from unknown organisms against a multi-genome database, or checking if DNA data have contamination from other organisms (Steinegger and Salzberg, 2020).

### 4.3 Words versus minimizers

This study indicates that seeding at minimally overlapping words is superior to minimizers. One caveat—bias due to reduced minimizer density at sequence edges—is addressed in the Supplementary Material, and does not change this conclusion. It is important to note, however, that minimizer schemes are still being optimized (Marçais *et al.*, 2017, 2018). On the other hand, we have barely begun to optimize word-restricted seeding.

Compared to word-restricted seeds, a minimizer seed match has an extra contextual requirement. A seed match can be destroyed by a mutation inside the seed: this applies equally to both methods. However, minimizers experience an additional effect: a mutation outside the seed can make that seed position no longer a minimizer. This reduces the sensitivity of minimizers, but increases their specificity, which fits our observations.

Our word-restricted seeding has a potential disadvantage: there is no upper bound on distance between words. The probability of longer distance decreases *exponentially* in complex sequence, but not in simple sequence, such as polypurine tracts or short-period tandem repeats. Pure simple-sequence similarities are typically not wanted, because their significance is hard to assess and they do not reliably indicate homology.

### 4.4 Further advantages of words

Word-restricted seeding has further advantages over minimizers. Firstly, it can be co-designed with subset seeds. Secondly, it seems likely that word positions can be found faster than minimizer positions. Thirdly, word-restricted seeding is more conducive to efficient indexes. Seed matches are usually found with an index data-structure. There are various kinds of index, but they often include a lookup table for any possible DNA sequence of some length *d*. This table can be reduced (or *d* increased) with word-restricted seeding, because only a subset of length-*d* words are ever considered.

### 4.5 Co-designed seed patterns

The sensitivity benefit of spaced and subset seeds can be enhanced by using, instead of one seed pattern, several co-designed patterns (Buhler *et al.*, 2003; Sun and Buhler, 2006). Each pattern tends to find similarities that tend to be missed by the other patterns. This idea could be combined with word-restricted seeding. For example, we could use four different patterns, each starting with one of the minimally overlapping words RRRY, RYRR, RYYR and YYYR (Table 3). Most interestingly, the best set of words may then not be minimally overlapping ones, but rather words whose overlaps complement the seed patterns.

### 4.6 Open questions

Our study provides a new motivation for the problem of maximizing the number of non-overlapping words. For our purposes, minimally overlapping words are especially useful, but we remain unsure how best to quantify overlap. Another challenge is how to search a large number of possible word sets for one with low overlap. More generally, we would like to design a set of word-restricted subset–seed patterns.

A further difficulty is how to optimize word-restricted seeding when the letter frequencies are unequal. In this case, we cannot simply seek an optimal set of *n* length-*k* words, because the sparsity is not constant. It is notable, however, that most natural DNA has near-equal frequencies of *r* and *y*.

Going further in the direction of empirical data, it might be useful to optimize word-restricted seeding for a particular sequence set (e.g. a genome). Presumably, it is beneficial to use words that are anti-clumped while tending to avoid repetitive sequence. Minimally overlapping words avoid some kinds of repeat, e.g. homopolymers. Previously, minimizer ordering was defined by frequency in a particular sequence set (Chikhi *et al.*, 2014).

Word-restricted seeding requires fast word-finding. Perhaps some word sets are conducive to fast detection, e.g. the eight length-7 words in Supplementary Table S1 share a common prefix.

When we use increasingly long minimum-variance words, with fixed sparsity *n*, the sensitivity might approach that of every-*n*th seeding (Figs 2 and 3). The seed count of every-*n*th seeding has zero variance: can the words achieve arbitrarily low variance? If so, they become arbitrarily close to a universal *k*-mer hitting set. Perhaps optimized minimizers, minimally overlapping words and universal *k*-mer hitting sets will converge.

## Supplementary Material

btaa1054_Supplementary_Data
